# Transcriptome analysis reveals the molecular mechanism of Yiqi Rougan decoction in reducing CCl_4_-induced liver fibrosis in rats

**DOI:** 10.1186/s13020-021-00552-w

**Published:** 2021-12-24

**Authors:** Yu Xiong, Jinyuan Hu, Chen Xuan, Jiayu Tian, Kaiyue Tan, Zhiwei Chen, Yan Luo, Xuqin Du, Junxiong Cheng, Lanyue Zhang, Wenfu Cao

**Affiliations:** 1 College of Traditional Chinese Medicine, Chongqing Medical University, No. 1 Medical College Road, Yuzhong District, Chongqing, 400016 China; 2 Chongqing Key Laboratory of Traditional Chinese Medicine for Prevention and Cure of Metabolic Diseases, Chongqing, 400016 China; 3 Department of Combination of Chinese and Western Medicine, The First Affiliated Hospital of Chongqing Medical University, Chongqing, 400016 China; 4 Department of Kidney Disease, Chongqing Traditional Chinese Medicine Hospital, Chongqing, 400021 China

**Keywords:** Yiqi Rougan decoction, Liver fibrosis, RNA sequencing, Endoplasmic reticulum stress, Apoptosis, Autophagy

## Abstract

**Background:**

Liver fibrosis develops from various chronic liver diseases, and there is currently a lack of specific treatment strategies. Yiqi Rougan decoction (YQRG) is a traditional Chinese medicine that has shown durative effects in the treatment of liver fibrosis; however, the mechanism associated with YQRG-related improvements in liver fibrosis remains to be experimentally determined. This study evaluated the therapeutic effect of YQRG on carbon tetrachloride (CCl_4_)-induced liver fibrosis in rats and its molecular mechanism.

**Methods:**

We used low-, medium-, and high-dose YQRG to treat CCl_4_-induced liver fibrosis in rats, followed by assessment of liver injury and fibrosis according to liver appearance, body weight, liver mass index, histopathologic examination, and serum testing. Additionally, we performed transcriptome analysis using RNA-sequencing (RNA-seq) technology, including cluster, Gene Ontology (GO), and pathway analyses, to identify differentially expressed genes (DEGs), and protein and gene expression were detected by immunofluorescence (IFC), western blot and real-time quantitative PCR.

**Results:**

The results showed that YQRG effectively alleviated CCl_4_-induced liver injury and fibrosis in rats, including observations of improved liver function, decreased activity of hepatic stellate cells (HSCs), and decreased extracellular matrix (ECM) deposition. Moreover, we identified downregulated and upregulated DEGs in the model group relative to the control and YQRG-treated groups, with GO analysis revealing their enrichment in biological processes, such as endoplasmic reticulum stress (ERS), apoptosis, and autophagy. Furthermore, pathway analysis showed that YQRG treatment downregulated the mitogen-activated protein kinase (MAPK) and phosphoinositide 3-kinase/Akt (PI3K/AKT) signalling pathways and upregulated other signalling pathways, including those related to peroxisome proliferator-activated receptors(PPAR) and AMP-activated protein kinase(AMPK), with these findings subsequently verified experimentally.

**Conclusion:**

These findings showed that YQRG improved CCl_4_-induced liver fibrosis through multiple mechanisms and pathways, offering critical insight into the YQRG-related therapeutic mechanism and promoting further research into its potential application.

**Supplementary Information:**

The online version contains supplementary material available at 10.1186/s13020-021-00552-w.

## Background

Hepatic fibrosis is an abnormal wound-healing process in which hepatic parenchymal cells (HPCs) transform into fibrous tissue of ECM [1–3], which is detrimental to human health. Liver fibrosis can subsequently evolve into liver cirrhosis and hepatocellular carcinoma, which account for 3.5% of annual global mortality [4]_._ It is difficult to accurately calculate the prevalence of liver fibrosis [5, 6], and it is generally believed that the condition is reversible. Therefore, actively reversing liver fibrosis is particularly important to the prevention and treatment of chronic liver disease. Notably, there is no specific anti-fibrosis therapy [7], making research supporting the active development of safe and effective anti-fibrosis drugs a significant undertaking to reveal their molecular mechanism.

Activation of resting hepatic stellate cells (HSCs) represents the critical mechanism associated with liver fibrosis formation [8–10]. Additionally, endoplasmic reticulum stress (ERS) describes the response to continuous aggregation of unfolded or misfolded proteins in the ER, which alters ER homeostasis [11], induces HPCs apoptosis, and reportedly promotes the formation of liver fibrosis [12, 13]. Moreover, apoptosis of HPCs, which can promote the transformation of HSCs from a static to an activated state, supports fibrotic initiation [14, 15]. Previous studies report that increased autophagy and levels of unfolded proteins can occur after the loss of cellular homeostasis, with these processes also implicated in the occurrence of fibrosis [16]. Furthermore, recent studies show that continuous ERS can activate autophagy, which subsequently promotes the activation of resting HSCs and induces liver fibrosis [17, 18].

Current treatment modalities for liver fibrosis mainly focus on eliminating the risk factors that are known to progressively develop into end-stage liver fibrosis, and treatment of end-stage liver fibrosis mainly involves liver transplantation, which carries the risk of trauma and rejection. Recently, the use of mesenchymal stem cell therapy for hepatic fibrosis has emerged, although there exists a risk of carcinogenesis. Therefore, it is particularly important to identify safe and effective anti-fibrosis therapies.

Traditional Chinese medicine has unique advantages in the treatment of chronic liver disease, especially in reducing hepatocyte damage, inhibiting inflammation, and promoting anti-fibrosis effects [19]. Yiqi Rougan (YQRG) decoction is a traditional Chinese medicine. Due to the complexity of traditional Chinese medicine prescriptions, the mechanism of action of YQRG in treating liver fibrosis remains unknown, including associated changes in gene expression. Transcriptome analysis using RNA-seq technology can potentially elucidate the molecular mechanism of disease occurrence [20]. In the present study, we analysed the main components of YQRG by ultra-high performance liquid chromatography with quadrupole time-of-flight mass spectrometry (UHPLC-QTOF-MS), evaluated its effects on liver function and fibrosis in a rat model of CCl_4_-induced liver fibrosis, and identified DEGs in rat liver following YQRG treatment.

## Methods

### Drugs and materials

YQRG was purchased from the Department of Traditional Chinese Medicine at the First Affiliated Hospital of Chongqing Medical University (Chongqing, China). CCl_4_ (batch NO. C805329) and olive oil (batch NO. O815210) were purchased from Shanghai Macklin Biochemical Co., Ltd. (Shanghai, China). Kits for assessing the levels of alanine aminotransferase (ALT; batch NO. S03030) and aspartate aminotransferase (AST; batch NO. S03040) were purchased from Shenzhen Rayto Life Technology Co., Ltd. (Shenzhen, China); hydroxyproline (HYP; batch NO. A030-3-1), from Nanjing Jiancheng Bioengineering Institute (Nanjing, China); and colchicine (batch NO. H20113208), from Guangdong Pidi Pharmaceutical Co., Ltd. (Guangdong, China). Enzyme-linked immunosorbent assay (ELISA) kits for hyaluronic acid (HA; batch NO. QZ-25723), laminin (LN; batch NO. QZ-25677), type IV collagen (IV-C; batch NO. QZ-25741), type III procollagen (PC-III; batch NO. QZ-25749), rat caspase 12 (CASP12; batch NO. QZ-20065), C/EBP homologous protein (CHOP; batch NO. QZ-25805), activated transcription factor 6 (ATF6; batch NO. QZ-25812), inositolase 1 (IRE1; batch NO. QZ-25833), phosphorylated (p)-extracellular signal-regulated kinase (PERK;batch NO. QZ-25829), and immunoglobulin-binding protein (BiP; batch NO. QZ-25818) were obtained from Quanzhou Jiubang Biotechnology Co., Ltd. (Quanzhou, China). Trizol (batch NO. 108–95-2), the Primescript RT kit, and gDNA eraser (batch NO. RR047A) were obtained from Takara Bio (Shiga, Japan). Primers for quantitative (q)PCR were provided by Tsingke Biotechnology (Beijing, China). Antibodies against α-smooth muscle actin (α-SMA; 19245), p38 (8690), p-p38 (4511), AMPK (2532), p-AMPK (2535), and β-actin (4970) were obtained from Cell Signaling Technology (Danvers, MA, USA). Antibodies against light-chain (LC) 3-I/II (ab128025) and LC3-II (ab192890) were obtained from Abcam (Cambridge, UK). Terminal deoxynucleotidyl transferase dUTP nick end labelling (TUNEL) staining kits (batch NO. G1501) were purchased from Wuhan Servicebio Biotechnology Co., Ltd. (Wuhan, China). Acetonitrile, methanol, and formic acid (LC–MS grade) were obtained from CNW Technology (Dusseldorf, Germany).

### YQRG preparation

YQRG contains eight herbs: *Astragalus mongholicus Bunge, Atractylodes Macrocephala Koidz, Salvia miltiorrhizae Bunge, Curcuma longa L, Paeonia lactiflora Pall, Cyperus rotundus L, Sargassum fusiforme Setch and Trichosanthes Kirilowii Maxim*. The herbal information and composition ratio are shown in Table 1. According to the surface area conversion ratio between rats and humans (6.3), we calculated the low-, medium-, and high-dose YQRG concentrations for use in rats at 4.95 g/kg, 9.9 g/kg, and 19.8 g/kg, respectively. According to this dosage, the Chinese herbs described were submerged in distilled water for 30 min, decocted three times, filtered, and concentrated three times to create YQRG decoctions at low, medium, and high dosages [21]. The composition of YQRG was determined by UHPLC-QTOF-MS for quality control.

**Table 1:** Composition of Yiqi Rougan decoction

Herbal medicine	Pinyin name	Batch number	Herb dose (g)	Occupied percent (%)
Astragalus mongholicus Bunge	Huang Qi	191,210,600	30	27.3
Atractylodes Macrocephala Koidz	Bai Zhu	200,310,099	10	9.1
Salvia miltiorrhizae Bunge	Dan Shen	200,201	15	13.6
Curcuma longa L	Jiang Huang	200,210,064	10	9.1
Paeonia lactiflora Pall	Bai Shao	200,210,085	15	13.6
Cyperus rotundus L	Xiang Fu	191,210,613	10	9.1
Sargassum fusiforme Setch	Hai Zao	190,701	10	9.1
Trichosanthes Kirilowii Maxim	Gua Lou	190,610,208	10	9.1

### UHPLC-QTOF-MS analysis

A methanol:water extract (4:1, v/v) was added to the YQRG decoction (150 µL) and vortexed for 30 s, followed by sonication for 1 h in an ice water bath and sitting at − 40 °C for 1 h and centrifugation at 12,000 rpm at 4° for 15 min. The supernatant was then passed through a 0.22-μm filter membrane for UHPLC tandem MS (MS/MS) analysis (Infinity 1290; Agilent Technology, Santa Clara, CA, USA). Mobile phases A and B comprised water with 0.1% formic acid and acetonitrile with 0.1% formic acid, respectively, and were passed at a flow rate of 400 µL/min. The sample (5 µL) was injected onto the Waters C18 column (1.7 × 2.1 µm × 100 mm; Waters Corp., Milford, MA, USA), followed by gradient elution: 0–3.5 min (5–15% B), 3.5–6 min (15–30% B), 6–12 min(30–70% B), 12–18 min (70–100% B), and 18–25 min (100% B). High resolution MS/MS was performed using a Q precision mass spectrometer (Thermo Fisher Scientific, Waltham, MA, USA), with the data analysed using Xcalibur software (Thermo Fisher Scientific).

### Animal experiments

Male Sprague–Dawley rats (n = 68; 180–220 g) were purchased from the Experimental Animal Center of Chongqing Medical University. Animal experiments were conducted in a specific pathogen-free animal laboratory (SYXK 2018–0003) at the Experimental Animal Center according to the Guidelines for the Care and Use of Experimental Animals from the National Institutes of Health (Bethesda, MD, USA). The experiments were approved by the Ethics Committee of Chongqing Medical University. After 1 week of adaptive feeding, the rats were randomly divided into two groups: the control (n = 8) and the CCl_4_-treatment group (n = 60). Rats in the CCl_4_-treatment group received intraperitoneal (i.p.) injection of 50% CCl_4_–olive oil solution (1 mL/kg) twice weekly for 9 weeks. At the end of the 4^th^ week, rats in the CCl_4_-treatment group were randomly divided into five experimental groups: low, medium, and high-dose treatment groups (n = 12) that received YQRG at 4.95 g/kg/day, 9.9 g/kg/day, and 19.8 g/kg/day, respectively; positive control group (n = 12) that received colchicine at 0.1 mg/kg/day; and the model group (n = 12) that received saline for 5 weeks. At the end of the 9th week, the rats were anaesthetized by i.p. injection of 2% pentobarbital sodium and asphyxiated with high-concentration CO_2_. Blood was collected from the abdominal aorta, the liver was removed immediately, and the weight was recorded. Liver tissue was quickly cut into pieces for subsequent liver haematoxylin and eosin (H&E) and Masson staining, immunohistochemistry (IHC), immunofluorescence staining, and transmission electron microscopy (TEM) analysis. The remaining tissues and serum were stored at −80 °C until further use.

### Liver index, Serum biochemical and liver HYP analyses

During the animal experiments, all rats were weighed and recorded every 2 weeks. Following liver removal and weighing, the liver index was calculated according to the body weight and the liver weight to evaluate liver injury, as follows: Liver index = (liver weight / body weight) × 100. According to the respective manufacturer instructions, Serum ALT, AST levels and liver HYP content were measured using kits. An automatic biochemical analyser (Shenzhen Rayto Life Technology Co., Ltd.) was used for the assays.

### Histologic analysis

Liver tissue was fixed with 4% paraformaldehyde for 24 h, dehydrated, embedded in paraffin, and sliced (4 µm), followed by staining with H&E and Masson Staining. For H&E staining, the slices were dewaxed first, soaked with hematoxylin dye solution for 10 min, and then washed with distilled water. After differentiation, the slices were rinsed again, and then rinsed with blue-returning solution. The slices were then soaked in ethanol (85% and 95%) for 5 min, and immersed in eosin dye for 10 min, followed by dehydration for 5 min (ethanol and xylene). Meanwhile, for Masson staining, the dewaxed slices were placed in Masson A for 15 h and rinsed, followed by soaking in the Masson mixture (Masson B:Masson C = 1:1) for 1 min, and washed. The mixture was differentiated with 1% hydrochloric acid alcohol for 1 min and washed. The tissue turned red after Masson D treatment for 6 min. The slices were rinsed, differentiated, dehydrated, and sealed with 1% glacial acetic acid. Eventually, tissue sections were observed and images were obtained using an optical microscope (BX53; Olympus, Tokyo, Japan).

### IHC analysis

Paraffin sections were dewaxed and soaked in citric acid antigen repair buffer to repair the antigen. The antigen was incubated with 3% hydrogen peroxide solution for 25 min, washed with phosphate-buffered saline (PBS), and sealed with 3% bovine serum albumin (BSA) at room temperature for 30 min. The antibody was incubated with anti-α-SMA and HRP-conjugated secondary antibodies, followed by DAB staining, rinsed, sectioned with differentiation solution, dehydrated with ethanol and xylene, and sealed with neutral rubber. These procedures were followed by washing, observation, and imaging using a microscope (BX53; Olympus).

### Assessment of ultrastructural morphology

The liver tissue was cut into 1 mm^3^ sections immediately upon collection of the liver (within 1–2 min), fixed in 2.5% glutaraldehyde, and washed with 1 M PBS. Subsequently, 1% osmic acid was added to the sections, which were incubated for 2 h, washed, dehydrated, penetrated, dehydrated with ethanol and acetone, and then embedded with 812 embedding agent. The embedding plate was polymerized for 48 h, the resin block was placed in the ultra-thin slicer (60 nm–80 nm), stained, washed, and dried with 2% uranium acetate saturated alcohol and 2.6% lead citrate solution, followed by observation of the ultrastructure of the liver and collection of images via TEM (JEM-1400plus; JEOL, Tokyo, Japan).

### RNA-seq analysis

RNA-seq was performed, and sequencing libraries were constructed using the Novaseq 6000 platform and the TruseqTM RNA sample prep kit (Illumina, San Diego, CA, USA). Total RNA was extracted with Trizol reagent, and mRNA was separated using oligo (DT) magnetic beads and randomly broken into small fragments. Six-base random primers were used to reverse transcribe cDNA, followed by end repair and sequencing. Changes in gene expression in liver tissue following YQRG treatment were assessed according to the identification of DEGs (fold changes > 2 or <  − 2; false discovery rate < 0.05). DEGs were clustered and analysed using the GO database and Kyoto Encyclopedia of Genes and Genomes (KEGG; http://www.genome.jp/kegg/) pathway analysis.

### Network construction and analysis

The STRING database (https://string-db.org/) is a database commonly used to predict protein–protein interaction (PPI). The main DEG was entered into the string database, the interaction score was set to ≥ 0.9, and the species was selected as "Homo sapiens" to create a PPI network. Then, the results were saved and exported in TSV format. Cytoscape (v.3.6.0; https://cytoscape.org/) was used to visualize the PPI networks. The TSV format file is imported into Cytoscape for PPI visualization.

### Double immunofluorescence staining

Double immunofluorescence staining was performed to detect colocalization of TUNEL staining and α-smooth muscle actin (a-SMA). Additionally, LC3-II and a-SMA were also examined. For the former staining, the slices were dewaxed in xylene, ethanol, and distilled water, and then circled into protease K working solution and incubated at 37℃ for 22 min, followed by washing with PBS, addition of 0.1% Triton, and incubation for 20 min at room temperature. Then, the buffer was incubated at room temperature for 10 min. TUNEL kit reaction solution (TDT enzyme:dUTP: Buffer = 1:5:50) was added into the ring and incubated for 2 h at 37 °C, and sealed with 3% BSA for 30 min. The sections were incubated with rabbit anti-α-SMA at 4 °C overnight, and then incubated with FITC-conjugated goat anti-rabbit IgG (Wuhan Servicebio Biotechnology Co., Ltd.) for 50 min at ambient temperature in the dark, followed by nucleus staining with 4',6-diamino-2-phenylindole (DAPI; Wuhan Servicebio Biotechnology Co., Ltd.) for 10 min. Additionally, For the latter staining, the sections were dewaxed, immersed in EDTA antigen repair buffer, circled, and sealed with 3% hydrogen peroxide and 3% BSA. Rabbit anti-LC3-II was added, and the sections were incubated overnight, followed by the addition of FITC-Conjugated goat Anti-Rabbit IgG, and incubation with Cy3-TSA for 10 min. The sections were then washed, and antigen repair was performed again, followed by incubation with the primary antibodies (anti-α-SMA), addition of FITC-conjugated goat anti-Rabbit IgG, and DAPI staining. The sections were then observed, and images were obtained by fluorescence microscopy (eclipse CI; Nikon, Tokyo, Japan).

### ELISA assay

ELISA was performed according to the manufacturer instructions. The levels of HA, LN, PC-III, IV-C, CASP12, CHOP, ATF6, IRE1, PERK and BiP in liver tissue were quantified. PBS was added to liver tissue for homogenization, followed by centrifugation at 4 °C for 20 min. This process was repeated three times, the supernatant was then used for ELISA detection, in which 50µL of termination solution was used and the absorbance was read at 450 nm, and finally the concentration of the appropriate substance was calculated.

### Real-time qPCR analysis

We then detected the mRNA levels of the genes *Acta2* (encoding α-SMA), *Hspa5* (encoding BiP), *Eif2ak3* (encoding PERK), *Atf6*, *Ern1* (encoding IRE1), *Chop*, *Casp12*, B-cell lymphoma-2 (*Bcl-2*), *Lc3b*, unc-51-like autophagy-activating kinase 1 (*Ulk1*), tissue inhibitor of metalloproteinase 1 (*Timp1*), metalloproteinase 9 (*Mmp9*), *P38*, and *Ampk*. Initially, 100 mg of liver tissue was weighed, added with 2 mL of trizol homogenate, and centrifuged at 12,000 rpm for 10 min, followed by the collection of 700 µL of supernatant to which 140 µL of chloroform (supernatant: chloroform = 1:0.2) was added, mixed well, and centrifuged. Subsequently, 300 µL of the supernatant was withdrawn, added with 300 µL isopropanol, and shook for 1 min. The liquid was then removed, 75% ethanol was added, washed, followed by addition of dH_2_O, and measurement and adjustment of the sample concentration. cDNA was synthesized by reverse transcription using a PrimeScript RT reagent kit with gDNA Eraser (Takara Bio). PCR amplification was then performed under the following cycling conditions: 95 °C pre-denaturation for 10 min, 95 °C denaturation for 15 s, 60 °C annealing for 1 min, for a total of 40 cycles (CFX96, Bio-Rad). *β-Actin* was used as the control for normalization of gene expression, and fold changes were calculated using the 2^–ΔΔCT^ method. The primer sequences are shown in Table 2.

**Table 2:** Primer sequences used for PCR

Gene	Oligonucleotide sequence (5′–3′)
*Acta2*	
Forward	AGACACCATGTGTGACGAGG
Reverse	GACCCATACCGACCATGACA
*Mmp9*	
Forward	CCAACCTTTACCAGCTACTCG
Reverse	TGAGTTCAATCCCCAGATGCC
*Timp1*	
Forward	CAAAGGATTCGACGCTGTGG
Reverse	TTCCGTTCCTTAAACGGCCC
*Atf6*	
Forward	CGCCGCAAGAAGAAGGAGTA
Reverse	CCTTCCTGTTTCCAGACCCC
*Ern1*	
Forward	CGATGGACTGGTGGTAACTG
Reverse	TGTCTCCTTGGGGAATGGAT
*Eif2ak3*	
Forward	GCTTGCTCCCACATCGGATA
Reverse	TGCGGCAATTCGTCCATCTA
*Hspa5*	
Forward	AGCCTGGTATGAGGATCTGC
Reverse	GACTGGAATCTGGAGAGCGA
*Chop*	
Forward	AGCCTGGTATGAGGATCTGC
Reverse	GACTGGAATCTGGAGAGCGA
*Casp12*	
Forward	AGGCCCATGTGGAGACAGAT
Reverse	GAGCCACTCTTGCCTACCTT
*Bcl2*	
Forward	CTGGTGGACAACATCGCTCT
Reverse	GCATGCTGGGGCCATATAGT
*Lc3b*	
Forward	CACAAGGGAAGTGATCGTCG
Reverse	AGCTGCTTCTCACCCTTGTA
*Ulk1*	
Forward	CATGGTCCCAGCCCAGTTTC
Reverse	TCGGAGAGGGAGATGGGGTA
*P38*	
Forward	AGATCAAGATCATTGCTCCTCCT
Reverse	ACGCAGCTCAGTAACAGTCC
*Ampk*	
Forward	AGATCAAGATCATTGCTCCTCCT
Reverse	ACGCAGCTCAGTAACAGTCC
*β-actin*	
Forward	AGATCAAGATCATTGCTCCTCCT
Reverse	ACGCAGCTCAGTAACAGTCC

### Western blot analysis

Proteins were extracted from liver tissue using a radioimmunoprecipitation buffer containing a protease inhibitor and phosphatase inhibitor and separated by electrophoresis, followed by transfer to polyvinylidene fluoride membranes. Membranes were washed five times with Tris-buffered saline containing Tween-20 (TBST) and incubated overnight at 4 °C with respective primary antibodies [α-SMA (1:1000), LC3-I/II (1:1000), p38 (1:1000), p-P38 (1:1000), AMPK (1:1000), p-AMPK (1:1000), and β-actin (1:1000)]. The membrane was then washed five times with TBST and incubated with HRP-conjugated secondary antibody (Boster Bio) for 1 h. Bands were visualized by enhanced chemiluminescence and images obtained using a chemiluminescence imaging system (Odyssey FC; Licor Biosciences, Lincoln, NE, USA). ImageJ software (National Institutes of Health, Bethesda, MD, USA) was used for data analysis.

### Statistical analysis

The data obtained are represented by the mean ± standard deviation. Statistical analysis was performed using SPSS 25.0 software (IBM, United States) (v.8.0.2; GraphPad Software, San Diego, CA, USA). One-way analysis of variance and a nonparametric test were used to compare the differences between groups. p < 0.05 was considered significant. Image synthesis was performed by the GraphPad Prism 8.0.2 software (San Diego, CA, United States).

## Results

### Component analysis of YQRG

**Fig. 1:**
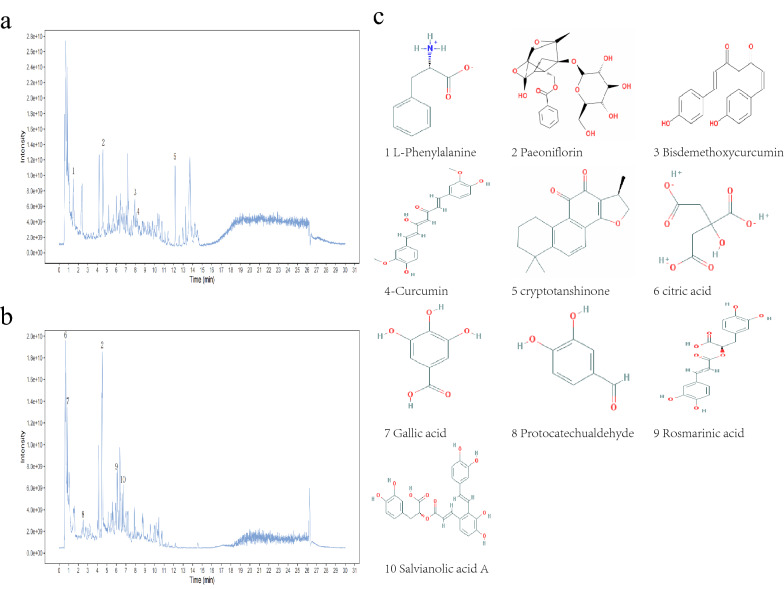
Identification of components of YQRG. Total ion chromatograms of positive (**a**) and negative (**b**) ion modes of YQRG samples were obtained using UHPLC–MS/MS. **c** Molecular structure of constituents

We analysed the main compounds of YQRG by UHPLC-MS/MS. Positive (Fig. 1a) and negative (Fig. 1b) ion chromatograms represented the YQRG compounds, revealing 10 main compounds (*L-Phenylalanine, Paeoniflorin, Bisdemethoxycurcumin, Curcumin, Cryptotanshinone, citric acid, Gallic acid, Protocatechualdehyde, Rosmarinic acid, Salvianolic acid A*) (Fig. 1c).

### YQRG improves liver injury in vivo

**Fig. 2:**
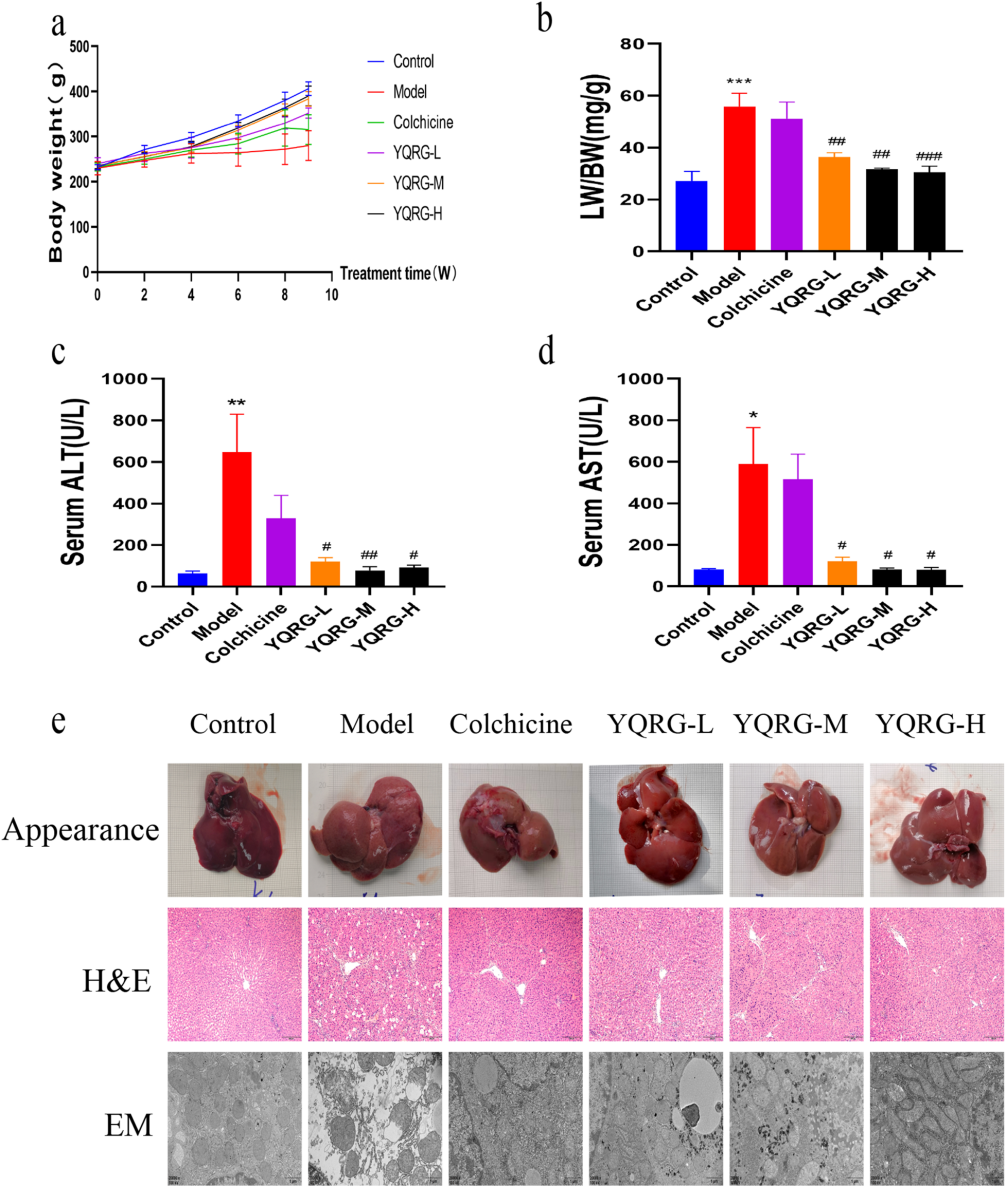
YQRG improves CCl_4_-induced liver injury in rats. **a** Body weight in different groups (n = 6). **b** LW/BW ratio in different groups. **c**, **d** Serum ALT and AST activities (n = 6). **e** Liver pathological changes were analysed by liver appearance (n = 6), H&E-staining (Scale bar = 200 μm, n = 6) of liver sections and transmission electron microscopy (Scale bar = 1 μm, n = 3) of rat livers. Data values are indicated as mean ± SD. *P < 0.05, **P < 0.01, ***P < 0.001, vs. control group; ^#^P < 0.05, ^##^P < 0.01, ^###^P < 0.001, vs. model group

To evaluate the effect of YQRG on liver fibrosis, we established a rat model of liver fibrosis according to the reported protocol [22]. During animal experiments, compared with the model group, the weight of the rats that received low, medium, or high concentrations of YQRG (YQRG-L, YQRG-M, and YQRG-H) or colchicine increased significantly (Fig. 2a). The liver weight ratio (LW: BW) used to evaluate liver injury showed that the LW: BW ratio decreased significantly in the YQRG treatment group (Fig. 2b). Next, we measured the levels of serum ALT and AST in rats to evaluate liver function. The results showed that the levels of ALT and AST in the model group increased significantly. Surprisingly, after YQRG treatment, the levels of ALT and AST decreased and were consistent with the normal levels (Fig. 2c and d). Then, we evaluated the morphological changes of cells in liver tissue from different angles by renal appearance, H&E staining and TEM. In the normal group, the liver looked ruddy and smooth, with sharp edges and a soft texture. In the rats that received CCl_4_, the appearance of the liver was lacklustre, the edge was blunt, and the surface was granular, rough, and hard. Compared with the model group, the livers of the YQRG group and the colchicine group were shinier, the edge was clearer, and the texture was soft and smooth (Fig. 2e). HE staining showed that the hepatocytes were arranged radially, centred on the central vein, and the structures of hepatic lobules and portal areas were clear. In the model group, the structure of hepatic lobules was damaged, the hepatocytes were degenerated and necrotic, and vacuolar changes and disordered arrangement appeared. In the YQRG-L, YQRG-M, YQRG-H, and the colchicine groups, the structure of hepatic lobules was improved and the vacuolar changes in hepatocytes were reduced and arranged neatly. Then, we observed the ultrastructure of the liver. Transmission electron microscopy analysis showed that the hepatocyte structure was damaged after CCl_4_ treatment, including mitochondrial swelling, endoplasmic reticulum damage, and bile duct dilatation. The tissue images of the YQRG groups and the colchicine group showed an improvement in pathological damage. The damage in hepatocyte structure was reduced, the swelling of mitochondria was improved, and the expansions of the endoplasmic reticulum and the bile duct were not obvious. These results suggested that YQRG can effectively resist the liver injury in rats induced by carbon tetrachloride.

### YQRG resists CCl_4_-induced liver fibrosis in vivo

**Fig. 3:**
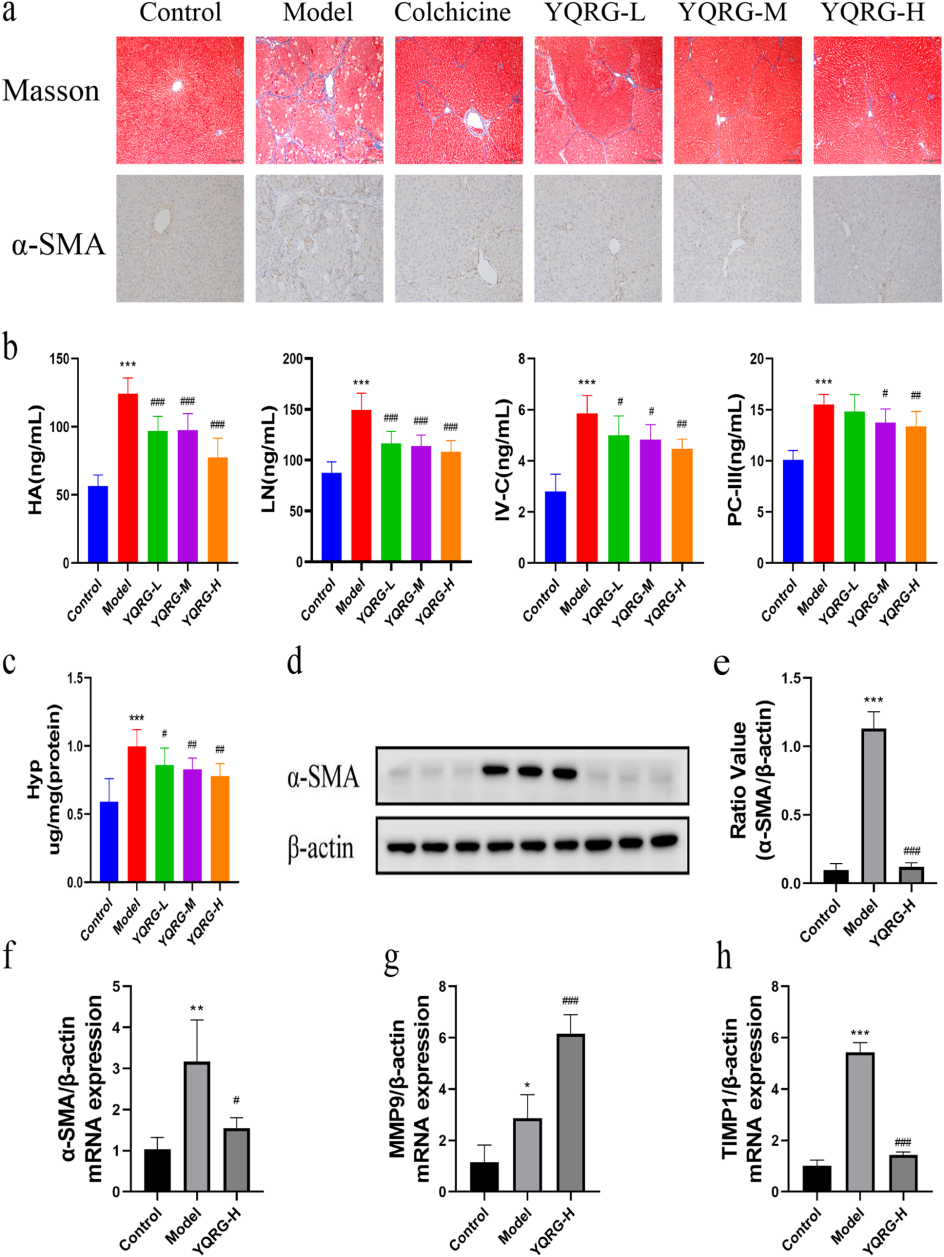
YQRG alleviates CCl_4_-induced liver fibrosis in rats. **a** Masson staining (Scale bar = 200 μm, n = 6) of rat livers and α-SMA were detected by immunohistochemistry (Scale bar = 100 μm, n = 6). **b** Serum HA, LN, PC-III, and IV-C content (n = 6). **c** HYP contents in liver tissues (n = 6). **d** Western blot image showing α-SMA expression in liver tissues (n = 6). **e** Protein concentration analysis (n = 6). **f**–**h** mRNA expression of α-SMA, MMP9, TIMP1 were detected by PCR (n = 6). Data values are indicated as mean ± SD. *P < 0.05, **P < 0.01, ***P < 0.001, vs. control group; ^#^P < 0.05, ^##^P < 0.01, ^###^P < 0.001, vs. model group

Next, we performed multiple experiments to evaluate the effect of YQRG against liver fibrosis. Masson staining showed that collagen fibres (blue) accumulated after CCl_4_ treatment, resulting in more fibre spacing, while collagen fibres and fibre spacing decreased in the YQRG groups and the colchicine group (Fig. 3a). Because HSCs activation promotes α-SMA expression, we evaluated liver injury and after treatment Changes in α-SMA levels. IHC results showed that the YQRG groups and the colchicine group reduced the positive expression of α-SMA relative to the model group (Fig. 3a). In addition, the fibrosis markers showed that the levels of HA, LN, PC-III, and IV-C in the model group were significantly higher than those in the YQRG groups (Fig. 3b). In addition, the level of HYP, the main component of collagen, increased in the model group but decreased after treatment in the YQRG groups (Fig. 3c). As shown in Fig. 3d–f, the results of α-SMA expression analysis support the results obtained in the immunohistochemical analysis, western blot, and qPCR analysis. Hepatic fibrosis is accompanied by an imbalance in ECM synthesis and degradation. ECM is regulated by TIMPs (promote ECM synthesis) and MMPs (promote ECM degradation) [23]. We analysed the mRNA expression levels of *TIMP1* and *MMP9* in the liver by qPCR. The results showed that, compared with the model group, the level of *MMP9* in the YQRF-H group increased significantly and the expression of *TIMP1* decreased significantly (Fig. 3g and h). These data show that YQRG-H processing reduces the accumulation of ECM. In “Conclusion”, these results suggest that YQRG-H treatment reduces CCl_4_-induced liver fibrosis in rats.

### Gene expression analysis by RNA-seq

**Fig. 4:**
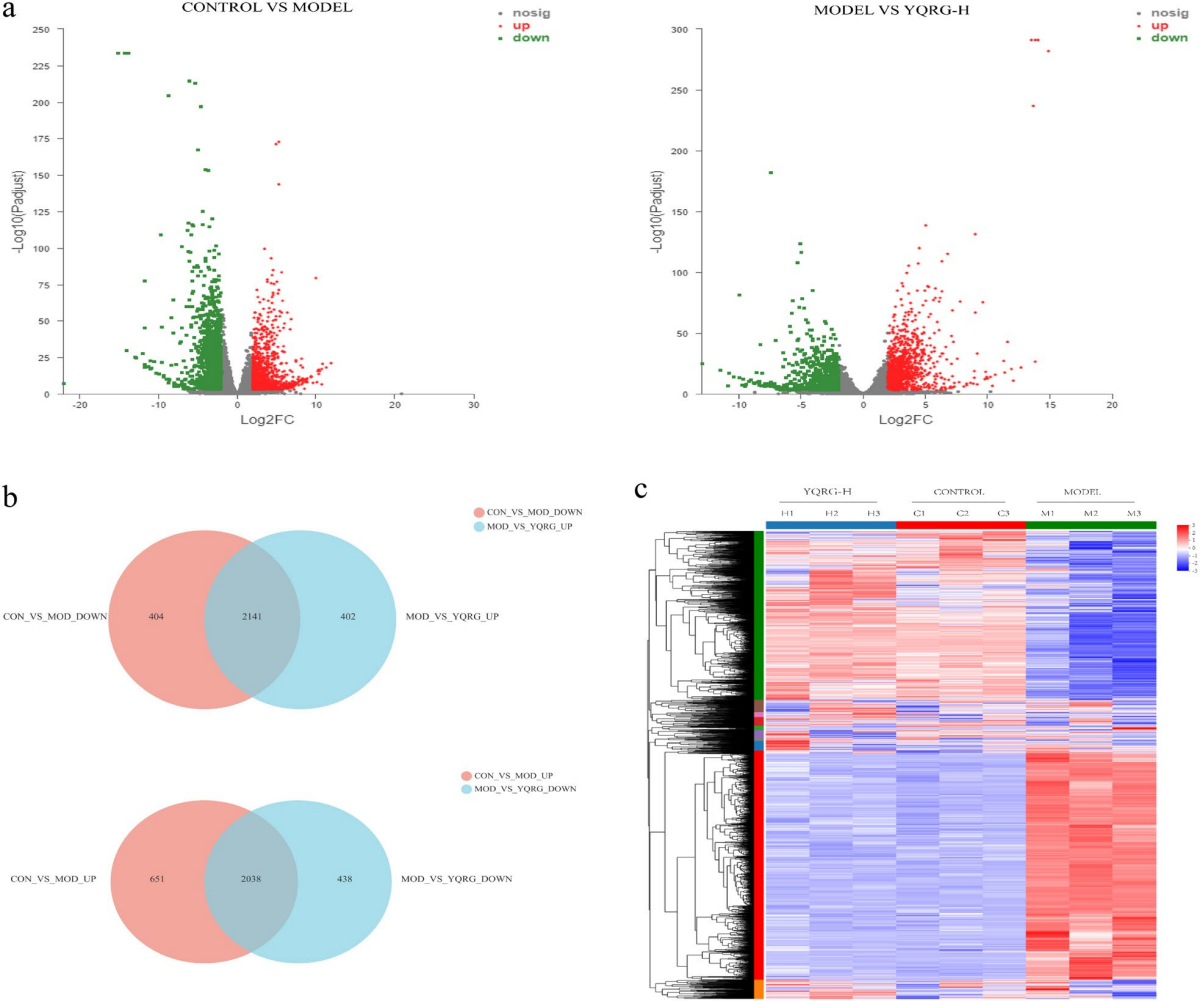
Gene expression analysis by RNA-seq. **a** Volcanic map of upregulated and downregulated DEGs between groups. **b** Venn diagram of the overlap of DEGs between groups. **c** Heat map for hierarchical cluster analysis of DEGs between samples

To gain insight into the molecular mechanism associated with the YQRG-mediated improvement in liver fibrosis in vivo, we performed RNA-seq analysis of samples from the control, model, and the YQRG-H-treated rats. We identified 2689 upregulated and 2545 downregulated DEGs in the control versus model groups, whereas the model versus YQRG-H groups revealed 2543 upregulated and 2476 downregulated DEGs (Fig. 4a). Compared with the normal control group and YQRG-H group, the upregulated and downregulated genes in the model group are the DEGs of YQRG in the treatment of CCl_4_-induced liver fibrosis, that is, 2141 YQRG upregulated DEGs and 2038 YQRG downregulated DEGs (Fig. 4b). Interestingly, these DEGs showed significant intergroup differences (Fig. 4c).

### GO/KEGG enrichment analysis and construction of PPI network

**Fig. 5:**
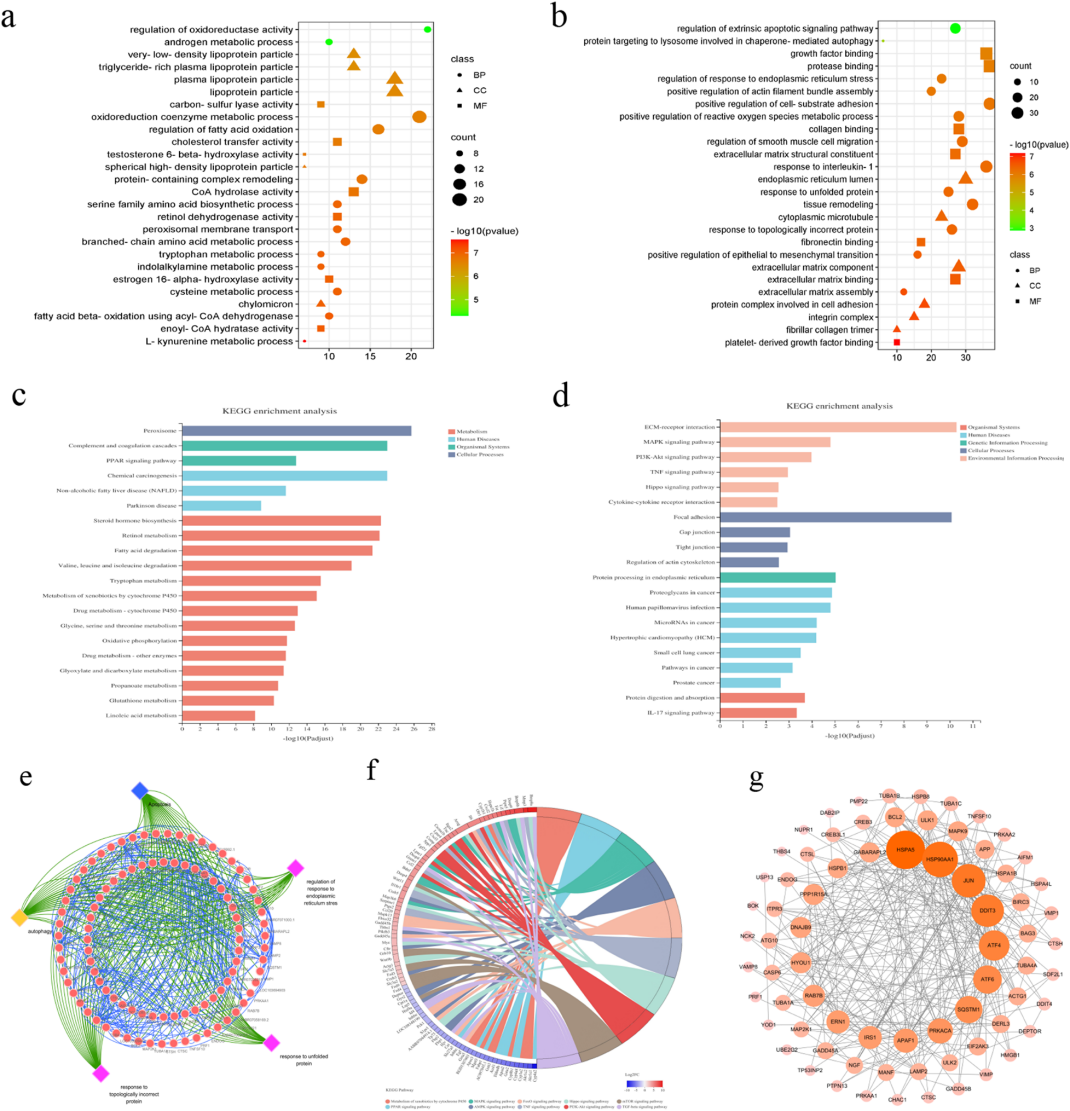
GO, KEGG analysis and PPI network. **a** and **b** GO enrichment analysis of 2141 overlapping upregulated DEGs and 2038 overlapping downregulated DEGs, including biological process (BP), cellular component (CC) and molecular function (MF). **c** and **d** KEGG enrichment analysis of 2141 overlapping upregulated DEGs and 2038 overlapping downregulatesd DEGs. **e** Interrelationships between genes involved in ERS, apoptosis, and autophagy. **f** Top 10 major KEGG signalling pathways chord involved in the anti-fibrosis effect of YQRG. Red indicated upregulated DEGs which all were upregulated in the model group relative to YQRG group. and blue indicated downregulated DEGs which all were downregulated in the model group relative to YQRG group. The closer log2FC is to the side, the greater the gene expression difference multiple. The closer log2FC is to the middle, the smaller the gene expression difference multiple. The KEGG pathway information on the enrichment of the first 10 genes with significant differences is shown below. **g** Visualization of PPI network by Cytoscape. circular nodes represent the major DEGs of YQRG; the size of the DEGs is directly proportional to their degrees in the network

After the DEGs of YQRG in the treatment of CCl4 induced liver fibrosis were obtained, we performed go and KEGG enrichment analysis for up-regulated and down-regulated DEGs respectively. GO enrichment analysis of the upregulated DEGs identified roles in biological processes (regulation of L-kynurenine metabolism and fatty acid oxidation, tryptophan metabolism, and branched-chain amino acid metabolism) (Fig. 5a). Downregulated DEGs were mainly involved in the biological processes involving positive regulation of epithelial-to-mesenchymal transition, response to topological error protein, response to unfolded protein, regulation of ERS, autophagy, and regulation of the apoptotic signalling pathway (Fig. 5b). KEGG pathway enrichment analysis showed that the upregulated DEGs were associated with the peroxisome, complement, and coagulation cascade; cytochrome P450 metabolism of exogenous substances; and peroxisome proliferator-activated receptor signalling (Fig. 5c), whereas the downregulated DEGs were associated with protein processing, MAPK signalling, PI3K/Akt signalling, and tumour necrosis factor signalling in the ER (Fig. 5d). Notably, GO analysis identified biological processes related to YQRG treatment of liver fibrosis were observed to be involved with ERS, apoptosis, and autophagy (Fig. 5e). The identified signal pathways and the related genes of the top 10 KEGG pathways closely related to liver fibrosis were plotted (Fig. 4f). The PPI network visualization were performed with the DEGs in biological processes such as ERS, apoptosis, and autophagy (Fig. 5g). The construction of the PPI network identified 33 target DEGs, as shown in Table 3.

**Table 3:** The major DEGs and their network degrees

Name	Degree	Name	Degree	Name	Degree
*Hspa5*	29	*Rab7b*	13	*Bag3*	9
*Hsp90aa1*	26	*Dnajb9*	12	*Birc3*	9
*Jun*	24	*Hyou1*	12	*Derl3*	9
*Ddit3*	23	*Ppp1r15a*	12	*Eif2ak3*	9
*Atf4*	21	*Bcl2*	11	*HSPA1B*	9
*Atf6*	19	*Gabarapl2*	11	*Tuba4a*	9
*Sqstm1*	18	*Hspb11*	11	*Gadd45a*	8
*Prkaca*	16	*Mapk9*	11	*Lamp2*	8
*Apaf1*	15	*Ulk1*	11	*Manf*	8
*Ern1*	14	*App*	10	*Ngf*	8
*Irs1*	14	*Actg1*	9	*Ulk2*	8

### YQRG treatment alleviates ERS in vivo

**Fig. 6:**
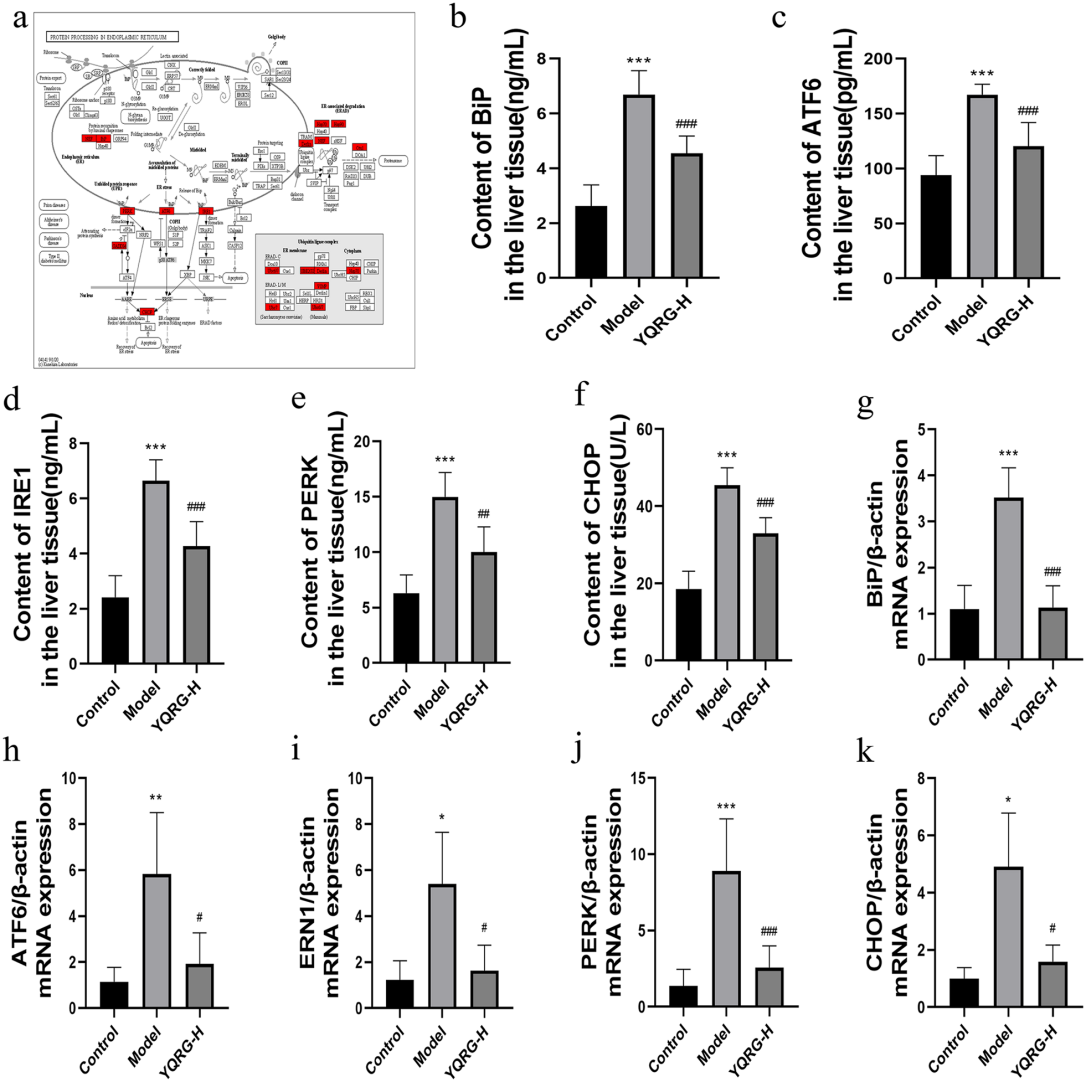
YQRG regulates ERS. **a** Mechanisms of ERS. **b**–**f** Levels of BiP, ATF6, IRE1, PERK, and CHOP were detected by ELISA in liver tissues (n = 6). **g**–**k** mRNA expression of *BiP*, *ATF6, ERN1, PERK, and CHOP* were detected by PCR (n = 6). Data values are indicated as mean ± SD. *P < 0.05, **P < 0.01, ***P < 0.001, vs. control group; ^#^P < 0.05, ^##^P < 0.01, ^###^P < 0.001, vs. model group

The inhibition of ERS is related to the improvement of liver fibrosis [24]. The formation mechanism of ERS is shown in Fig. 6a. Transcriptome analysis of the GO and KEGG pathways showed that they were enriched in endoplasmic reticulum stress-related genes and pathways. BiP, PERK, ATF6, IRE1, and CHOP are considered to be important markers of ERS [24]. To evaluate the effect of YQRG treatment on ERS, we detected the changes in the expression of ERS-related markers BiP, ATF6, PERK, IRE1, and CHOP by ELISA and qPCR. Consistent with the RNA-seq results, BiP, PERK, ATF6, IRE1, and CHOP protein and mRNA levels in the model group were significantly higher than those in the control group, indicating that CCl_4_ administration induced ERS. However, YQRG-H treatment reversed these changes, resulting in levels similar to those in controls (Fig. 6b–k), and leading to reductions in ERS. These findings suggested that YQRG-H treatment effectively alleviated ERS.

### YQRG treatment regulates cell apoptosis

**Fig. 7:**
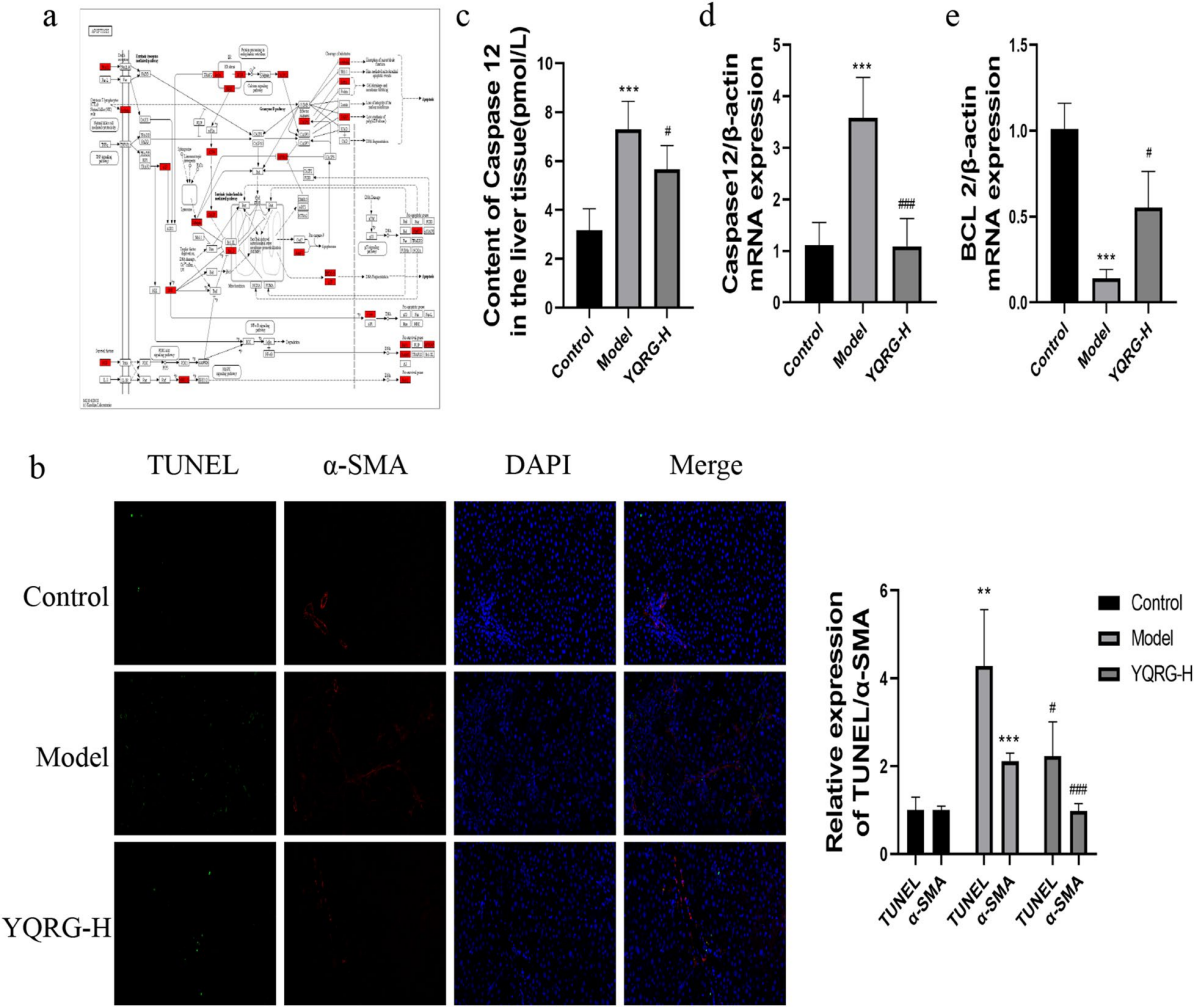
YQRG regulates apoptosis. **a** Mechanisms of Apoptosis. **b** TUNEL and α-SMA double immunofluorescence staining in liver tissues (Scale bar = 50 μm, n = 3), green fluorescence indicated the apoptosed cells, red fluorescence represented α-SMA. **c** Levels of caspase 12 were detected by ELISA in liver tissues (n = 6). **d**, **e** mRNA expression of *caspase 12* and *Bcl2* were detected by qPCR (n = 6). Data values are indicated as mean ± SD. *P < 0.05, **P < 0.01, ***P < 0.001, vs. control group; ^#^P < 0.05, ^##^P < 0.01, ^###^P < 0.001, vs. model group

Cellular apoptosis promotes the occurrence of liver fibrosis, and this process is very complex (Fig. 7a). We performed TUNEL and α-SMA (a marker for the activation of HSCs [25]) double immunofluorescence staining in liver tissues to evaluate the effect of YQRG treatment on cellular apoptosis. The results showed an increased number of apoptotic cells in the model group relative to the control group along with a significant increase in the expression of α-SMA. However, YQRG-H treatment significantly reduced the number of apoptotic cells and a-SMA expression levels in the model group (Fig. 7b). Next, the expression of apoptosis-related proteins in liver tissue was detected by ELISA and qPCR. CASP12 and BCL-2 are apoptosis-related markers and play an important role in apoptosis [26]. From the ELISA and qPCR analysis, it was observed that, in the CCl_4_-induced group, the expression of CASP12 increased significantly. In contrast, YQRG-H treatment decreased the expression level of CASP12 (Fig. 7c, d). Similarly, compared with the model group, the expression of BCL-2 in the YQRG-H group increased (Fig. 7e). These results suggested that YQRG-H treatment inhibits apoptosis.

### YQRG treatment regulates autophagy in vivo

**Fig. 8:**
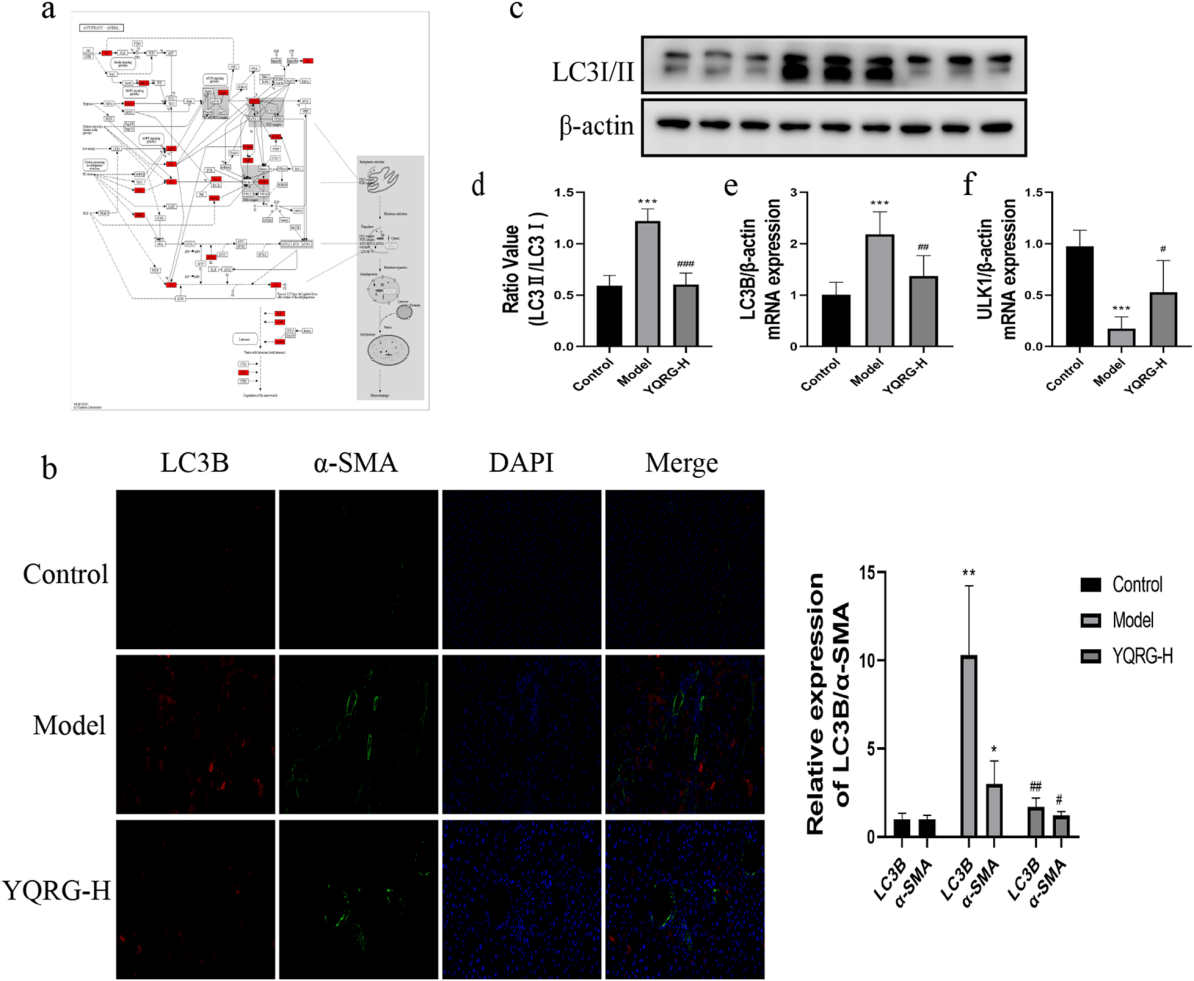
YQRG regulates autophagy. **a** Mechanisms of autophagy. **b** Double immunofluorescent staining was performed to determine the colocalization of LC3II (red) and α-SMA (blue) in liver tissues (Scale bar = 50 μm, n = 3). **c** Western blot image showing LC3I/II expression in liver tissues (n = 6). **d** Protein concentration analysis (n = 6). **e**, **f** mRNA expression of *LC3B* and *ULK1* were detected by qPCR (n = 6). Data values are indicated as mean ± SD. *P < 0.05, **P < 0.01, ***P < 0.001, vs. control group; ^#^P < 0.05, ^##^P < 0.01, ^###^P < 0.001, vs. model group

Autophagy is a metabolic process that is closely related to liver fibrosis (Fig. 8a). To determine whether YQRG-H treatment affects autophagy, we evaluated the autophagic flux by double immunofluorescence staining, western blot, and qPCR. LC3-II is a marker for autophagy [27], and double immunofluorescence staining of LC3-II and α-SMA revealed that YQRG-H treatment reduced the fluorescence signals of LC3-II and α-SMA in the model group (Fig. 8b). This result suggested that autophagy activation was closely related to HSCs activation in the model group. Additionally, western blot and qPCR analyses confirmed the increased expression of the LC3-II protein and mRNA in the model group, whereas these levels were decreased following YQRG-H treatment (Fig. 8c–e). ULK1 is also an important indicator that regulates autophagy [28], and qPCR analysis showed that the expression of *Ulk1* was significantly lower in the model group than in the control group and the YQRG-H group (Fig. 8f). These results suggested that CCl_4_ can promote autophagy and that YQRG treatment inhibits this process.

### Effects of YQRG treatment on cell signalling pathways

**Fig. 9:**
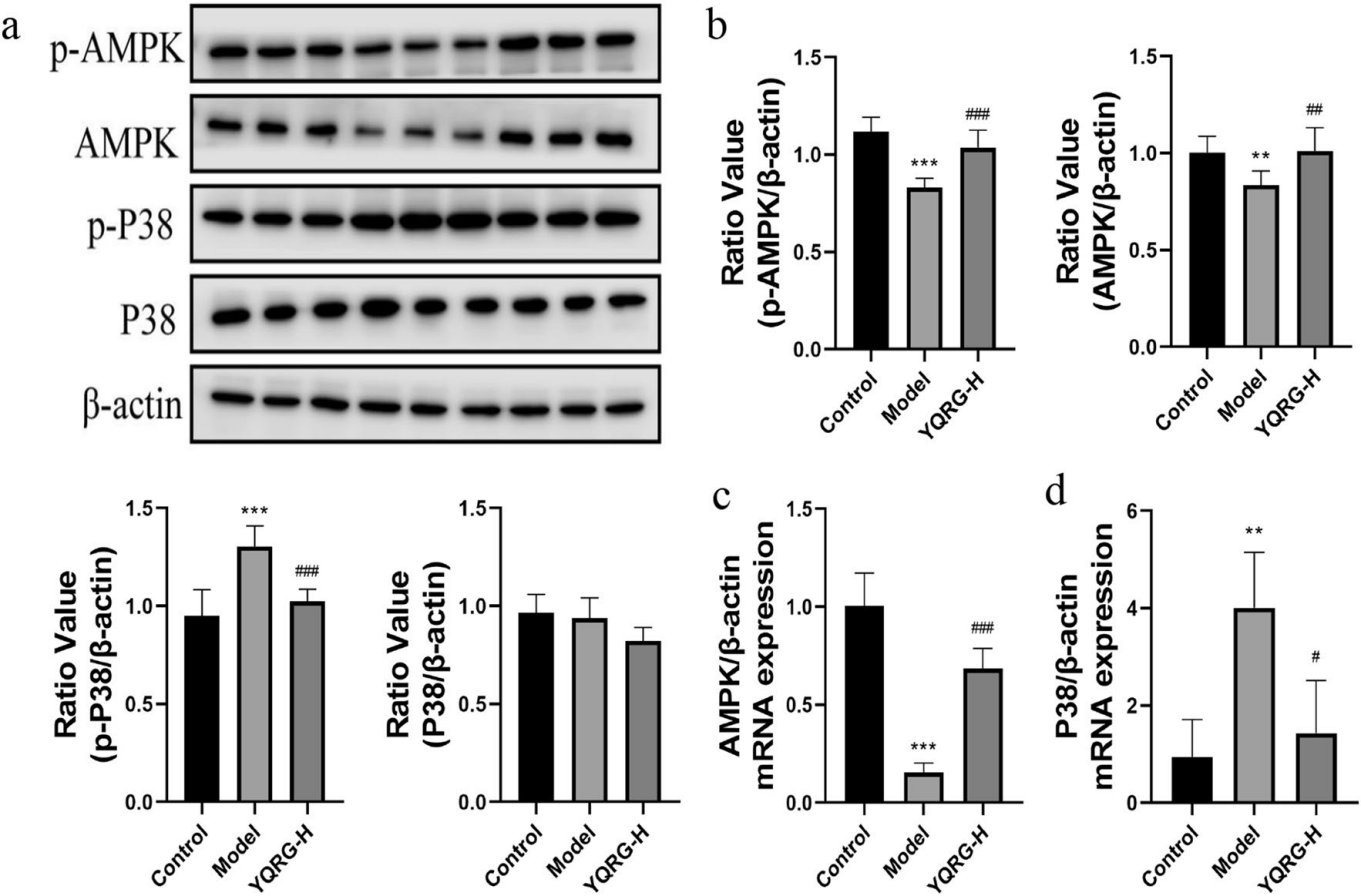
Effect of YQRG on the P38 MAPK and AMPK signaling pathways. **a** Protein expressions of P38、p-P38、AMPK and p-AMPK were determined by Western blot (n = 6). **b** Protein concentration analysis (n = 6). **c**, **d** mRNA expression of *P38, AMPK* were detected by PCR (n = 6). Data values are indicated as mean ± SD. *P < 0.05, **P < 0.01,***P < 0.001, vs. control group; ^#^P < 0.05, ^##^P < 0.01, ^###^P < 0.001, vs. model group

To further evaluate the mechanism associated with the YQRG treatment of liver fibrosis, we evaluated the markers related to the pathways identified by KEGG analysis. Western blot analysis of liver tissue samples from the control, model, and YQRG-H groups revealed the expressions of P38, p-P38, AMPK, and p-AMPK, with upregulated levels of p-P38 and downregulated levels of AMPK and p-AMPK observed in the model group compared to the control group. Following the YQRG-H treatment, we observed significantly decreased p-P38 levels and increased AMPK and p-AMPK levels in the YQRG-H compared to the model group (Fig. 9a and b). Moreover, qPCR results were consistent with the results obtained from western blot analysis (Fig. 9c and d). These results suggested that YQRG-H treatment inhibited P38-MAPK signalling and activated AMPK signalling.

## Discussion

Hepatic fibrosis is a repair response to chronic liver injury. Due to the proliferation of myofibroblasts under the action of different stimuli, hepatocytes initiate the production of various chemokines, which promote myofibroblast proliferation in the liver injury area, resulting in the formation of collagen and other fibre components, ECM accumulation, and fibrosis. Liver injury from various causes leads to liver fibrosis, which is potentially followed by cirrhosis and liver cancer. There is currently a lack of therapeutic options for the prevention of hepatic fibrosis, making it necessary to research and actively develop effective drugs. YQRG, a traditional Chinese medicine comprised of eight herbs, is a mixture of compounds. In this study, we identified YQRG compounds by UHPLC-QTOF-MS (Additional file 1), most of which were previously identified as playing a role in improving liver fibrosis (i.e., *Paeoniflorin, Bisdemethoxycurcumin, Curcumin, Cryptotanshinone, Gallic acid, Protocatechualdehyde, Rosmarinic acid, Salvianolic acid A*) [29–36] (Fig. 1c). This result suggested that most of the sampled compounds may be the components of YQRG which mitigate liver fibrosis.

The liver can be damaged by infection, excessive drinking, or other factors [37], and various liver injuries can promote the expression of fibrogenic mediators and their receptors in response to liver fibrosis [38]. Because CCl_4_ exerts hepatotoxicity, its administration can seriously damage hepatocytes [39]. Liver injury is reversible upon the removal of the stimuli [40]; therefore, numerous studies have evaluated the mechanisms associated with CCl_4_-induced liver injury. In this study, we evaluated the effect of YQRG on rat models of CCl_4_-induced liver injury. The body weight significantly increased (Fig. 2a) and the LW:BW ratio decreased in the YQRG groups and the colchicine group (Fig. 2b). Moreover, we measured the serum ALT and AST levels (Fig. 2c and d) as markers of hepatocyte integrity [41], and observed significantly increased levels of both enzymes in the model group, whereas these levels were restored to levels similar to that in the control group after YQRG treatment. Additionally, H&E staining and TEM analysis (Fig. 2e) revealed evidence of pathological hepatocyte injury in the model group; however, these characteristics were significantly improved following YQRG treatment and colchicine treatment. These results indicated that YQRG treatment effectively reduced CCl_4_-induced liver injury.

YQRG has been used to clinically treat liver fibrosis efficiently; however, there is currently a lack of experimental data supporting its efficacy. Here, we established a rat model of liver fibrosis to evaluate the efficacy and mechanism of YQRG against liver fibrosis induced by CCl_4_ [42]. At present, the gold standard for the clinical diagnosis of liver fibrosis is liver biopsy requiring sections of at least six portal triads. According to METAVIR, liver fibrosis, as observed from liver biopsies, is categorized into five groups namely F0 to F4 (F0 = no fibrosis; F1 = portal fibrosis without septa; F2 = portal fibrosis with few septa; F3 = numerous septa without cirrhosis; F4 = cirrhosis) [43]. H&E (Fig. 2e) and Masson staining (Fig. 3a) showed that the structure of hepatic lobules in the control group was normal, the hepatocytes were arranged orderly, and few collagen fibres existed, but within the normal range (METAVIR F0). In the model group, the hepatic lobule structure was damaged, the hepatocytes were arranged disorderly, a large number of collagen fibres were deposited in the portal area, and the collagen fibres were connected to form a fibre septum (METAVIR ≥ F2), indicating that the rats had fibrosis. Although different degrees of collagen fibres in the portal area were observed in the YQRG-treated group and the colchicine group, the number of collagen fibres and the fibre septum decreased (METAVIR F1-F2). This showed that the YQRG-treated group and the colchicine group had significantly reduced fibrosis. Additionally, we evaluated the serum levels of the fibrotic markers HA, LN, PC-III, and IV-C [44]. HYP, an important component of collagen tissue, comprises up to 13% of collagen [45] and represents another marker of liver fibrosis [46]. In the present study, the levels of serum HA, LN, PC-III, IV-C (Fig. 3c), and HYP (Fig. 3d) in liver tissue were significantly higher in the model group than in the control and the YQRG-treatment groups. Moreover, in liver fibrosis, excessive accumulation of ECM is mainly due to HSC activation [8], and we observed significant increases in α-SMA (a marker of HSCs activation [25]) levels, detected by IHC (Fig. 3a), western blot (Fig. 3d) and qPCR (Fig. 3f), in the model group, while this level decreased after YQRG-H treatment. ECM is regulated by TIMPs and MMPs [23], and a previous study has reported that inhibiting HSC activity can reduce TIMPs secretion and increase MMPs activity, thereby promoting ECM degradation [47]. Consistent with these findings, in the present study, we found that YQRG-H treatment significantly reduced *Timp1* expression and increased *Mmp9* expression in the model group (Fig. 3g and h). These findings suggested that YQRG treatment effectively improved CCl_4_-induced liver fibrosis in rats by attenuating HSCs activity and inhibiting ECM synthesis.

ER is the largest organelle in HPCs and exerts the functions of participating in protein folding and regulating calcium homeostasis [48, 49]. The stimulation of various factors leads to the destruction of ER, promotes the accumulation of misfolded proteins, and then activates the unfolded protein response (UPR) [50, 51]. ATF6, IRE1, and PERK are transmembrane sensors of UPR [52], and ERS is closely related to the increased expression of the UPR-related genes. For instance, Zhan et al. has reported that microcystin-LR can up-regulate the gene levels of PERK and IRE1 in the liver and the ovary, causing ERS [53]. In this study, the levels of liver ATF6, IRE1, and PERK in ELISA and the qPCR analysis increased after CCl4 treatment (Fig. 6c–e and h–j). Under stress-free conditions, BiP, an important member of the heat shock protein 70 family, binds to members of the UPR. Under ERS, BiP binds to members of the wrong or unfolded proteins and separates from UPR, activates these sensors and their downstream signal cascade through dimerization and autophosphorylation, and promotes the apoptosis regulator CHOP [54]. In our study, the levels of BiP and apoptotic transcription factor CHOP in the liver of rats with hepatic fibrosis were higher than those in the YQRG-H group in the ELISA snalysis (Fig. 6b and f, g and k). The Study has shown that inhibiting ERS can reduce liver fibrosis [55]. In this study, transcriptome analysis showed that ERS inhibition was an important mechanism of the YQRG-mediated reduction of CCl_4_ induced liver fibrosis (Fig. 5b and d). This is consistent with our experimental data and transcriptome analysis results, suggesting that YQRG treatment reduces CCl_4_-induced liver fibrosis in rats by inhibiting ERS.

HPCs apoptosis is an inflammatory stimulus that promotes HSCs and plays an important role in liver diseases [56, 57]. We observed a significant increase in TUNEL and α-SMA positive cells in the liver of rats in the CCl_4_ group (Fig. 7b).Under sustained ERS, pro-apoptotic signals are induced by activating several transcription factors [58, 59]. CHOP is a key transcription factor related to the ERS-mediated activation of HPCs apoptosis [60]. Under continuous ERS, the pro-apoptotic factor CHOP inhibits the downstream anti-apoptotic protein BCL-2. In addition, Ca^2+^ enters the cytoplasm, m-calpain and procaspase 12 are cleaved and activated, and the caspase cascade induces apoptosis [61]. Apoptosis occurs mainly through endogenous and exogenous apoptosis pathways. In the endogenous apoptosis pathway, mitochondrial cytochrome c is released, caspase 9 and caspase 3 are activated in order. In the exogenous apoptotic pathway, caspase 3 is activated by upstream bound caspase 8 and by fas-associating protein with a novel death domain (FADD). Death receptors such as TNF related apoptosis inducing ligand (TRAIL) bind to FADD to further activate downstream caspase 8 and caspase 3, and caspase 3 is the co-executor of the two pathways [62–65]. In this study, we demonstrated the expression of CHOP, BCL2, and caspase 12 in fibrotic rats (Figs. 6f and k, 7c–e), which implied that apoptosis occurs during the development of fibrosis. These results, when combined with the significant increase in ERS related indexes in the model group, suggested that CCl_4_ may induce apoptosis by activating ERS.

Autophagy is a metabolic process in which autophagosomes formed by cell membranes phagocytize organelles and cell fragments for subsequent lysosomal degradation [66]. Studies have shown that ERS-mediated autophagy in HSCs promotes the occurrence of liver fibrosis. For instance, Men R et al. reported that after NOGO-B gene knockout, the levels of ERS and autophagy markers were down-regulated and the autophagy level could be regulated by ERS agonists and antagonists. This study results showed that inhibiting ERS could reduce the autophagy of HSCs [67]. Furthermore, overexpression of *Ulk1* kinase-dead mutant can inhibit autophagy [28], and LC3-II acts as an autophagy marker [27]. Consistently, we found that prolonged exposure to CCl_4_ had an impact on the expression of two key autophagy genes (*LC3B* and *ULK1*). In the present study, the number of LC3-II and α-SMA positive cells increased significantly (Fig. 8b), indicating that the increased autophagy in the liver fibrosis model is related to HSCs activation. The protein and mRNA levels of LC3II increased in the model group and the mRNA expression of *Ulk1* decreased in the model group (Fig. 8c–f). These results suggested that YQRG improves liver fibrosis by inhibiting autophagy.

Pathway analysis suggested that YQRG treatment altered the MAPK and the AMPK signalling pathways. P38 MAPK plays an important role in regulating inflammation, apoptosis, and liver fibrosis [68, 69], and a previous study has reported that ERS activates P38 MAPK signalling [70]. Additionally, ERS-mediated IRE1/P38 MAPK signalling promotes the activation of HSCs in rats [71]. In the present study, we found that YQRG treatment inhibited P38 MAPK signalling (Fig. 9a and d) along with the expression of ERS-related markers, suggesting that YQRG might alleviate liver fibrosis by inhibiting ERS through regulation of P38 MAPK signalling. Moreover, P38 MAPK promotes apoptosis [72] and is associated with autophagy induction. AMPK is an energy sensor that plays an important role in maintaining energy homeostasis [73]. A previous study showed that AMPK exerts an anti-apoptotic effect and can inhibit HPCs apoptosis [74]. Additionally, AMPK activation inhibited autophagy in an HSC line (lx-2) [31]. In the present study, YQRG treatment promotes AMPK signalling (Fig. 9a and d). Moreover, the observed regulation of apoptosis by YQRG suggested that the activation of the AMPK pathway might inhibit apoptosis and autophagy, thereby alleviating the progression of liver fibrosis. However, the precise role of YQRG as a therapeutic agent for liver fibrosis requires further investigation.

## Conclusion

In conclusion, as inferred from transcriptome analysis and experimental verification, YQRG can inhibit CCl_4_-induced liver fibrosis through a variety of mechanisms. This study showed that YQRG improved liver pathological injury and function, inhibited HSC activity, and reduced ECM accumulation in rats with liver fibrosis. The anti-liver fibrosis effect of YQRG might be related to the inhibition of ERS, apoptosis, and autophagy and the regulation of P38 MAPK and AMPK pathways.

## Supplementary Information

**Additional file 1.** Detailed information of YQRG-compounds were obtained by MS/MS.

## Data Availability

I have uploaded the original sequential data in public database of SRA under accession number PRJNA781107.

## Abbreviations

α-SMA: α-Smooth muscle actin
ALT: Alanine aminotransferase
AMPK: AMP-activated protein kinase
AST: Aspartate aminotransferase
ATF6: Activated transcription factor 6
BCL-2: B-cell lymphoma 2
BiP: Immunoglobin-binding protein
BP: Biological process
BSA: Bovine serum albumin
CASP12: Caspase 12
CC: Cellular component
CCl_4_: Carbon tetrachloride
CHOP: C/EBP homologous protein
DAPI: 4′,6-Diamino-2-phenylindole
DEG: Differentially-expressed gene
ECM: Extracellular matrix
ELISA: Enzyme-linked immunosorbent assay
ERS: Endoplasmic reticulum stress
FADD: Fas-associating protein with a novel death domain
GO: Gene Ontology
H&E: Haematoxylin and eosin
HA: Hemagglutinin
HPCs: Hepatic parenchymal cells
HSCs: Hepatic stellate cells
HYP: Hydroxyproline
IFC: Immunofluorescence
IHC: Immunohistochemistry
i.p.: Intraperitoneal
IRE1: Inositolase 1
IV-C: Type IV collagen
KEGG: Kyoto Encyclopedia of Genes and Genomes
LN: Laminin
LC: Light chain
LW:BW: Liver-to-body weight ratio
MAPK: Mitogen-activated protein kinase
MF: Molecular function
MMP9: Matrix metalloproteinase 9
PBS: Phosphate-buffered saline
PC-III: Procollagen III
PERK: Protein kinase R-like ER kinase
PI3K/AKT: Phosphoinositide 3-kinase/Akt
PPAR: Peroxisome proliferator-activated receptors
PPI: Protein–protein interaction
qPCR: Quantitative PCR
RNA-seq: RNA sequencing
TBST: Tris-buffered saline with Tween-20
TEM: Transmission electron microscopy
TIMP1: Tissue inhibitor of metalloproteinase 1
TRAIL: TNF related apoptosis inducing ligand
TUNEL: Terminal deoxynucleotidyl transferase dUTP nick end labelling
UHPLC- QTOF-MS: Ultra-high performance liquid chromatography with quadrupole time-of-flight mass spectrometry
ULK1: Unc-51-like autophagy-activating kinase 1
UPR: Unfolded protein response
YQRG: Yiqi Rougan decoction
